# A Step-Wise Approach to Total Laparoscopic Gastrectomy with Jejunal Pouch Reconstruction: How and Why We Do It

**DOI:** 10.1007/s11605-016-3235-7

**Published:** 2016-08-25

**Authors:** Hylke J. F. Brenkman, Juan Correa-Cote, Jelle P. Ruurda, Richard van Hillegersberg

**Affiliations:** 1 Department of Surgery, University Medical Center Utrecht, PO BOX 85500, 3508 GA Utrecht, The Netherlands; 2 Department of Surgical Oncology, Hospital Pablo Tobón Uribe, Calle 78 B #, 69 - 240 Medellín, Colombia; 3 Department of Surgical Oncology, University of Toronto, Room 3-130, 610 University Avenue, Toronto, ON M5G 2M9 Canada

**Keywords:** Laparoscopy, Gastrectomy, Jejunal pouch, Gastric cancer

## Abstract

Laparoscopic gastrectomy (LG) is a safe alternative compared to open gastrectomy for cancer. To increase the uptake of minimally invasive approaches and facilitate their analysis and improvement a stepwise approach is warranted. This study describes our technique and experiences total laparoscopic gastrectomy (TLG) with jejunal pouch reconstruction for gastric cancer. Technical modifications throughout the years were described. In patients with anastomotic leakage, the CT-scan and reoperation report were reviewed to identify the location and cause of the leak. A total of 47 patients who underwent laparoscopic total gastrectomy with extracorporeal jejunal pouch reconstruction and stapled circular esophagojejunostomy from May 2007 to August 2015 were prospectively analyzed. A stepwise approach of 10 steps was designed based on video and case analysis. Median operation time was 301 (148–454) minutes and median blood loss was 300 (30–900) milliliters. Anastomotic leakage occurred in six (12.8 %) patients; additionally, one (2.12 %) jejunal-pouch staple line leak was identified. An important modification in our technique was a purse-string suture around the anvil of the circular stapler to prevent esophageal mucosa to slip away. After this modification, the leakage rate was reduced to 7 % in the last 15 procedures. In conclusion, TLG with jejunal pouch reconstruction is a feasible procedure in a selected group of patients. Our stepwise approach and technique may help surgeons to introduce jejunal pouch reconstruction during laparoscopic gastrectomy in their center.

**Electronic supplementary material:**

The online version of this article (doi:10.1007/s11605-016-3235-7) contains supplementary material, which is available to authorized users.

## Introduction

Gastric cancer (GC) is the fifth most common malignancy worldwide, and the third leading cause of cancer-related death.^1^^,^ 2 In the era of improved outcomes with a multidisciplinary approach, surgery continues to be the cornerstone of treatment.^3^ Minimally invasive gastrectomy (MIG) has been proven to be a safe alternative in Distal Gastrectomies (DG),^4^^–^ 8 showing decreased length of stay, less perioperative complications, less bleeding at the cost of increased operating room (OR) time. The safety of total laparoscopic gastrectomies (TLG) has also been studied recently by our group and others,^9^^–^ 11 with similar advantages to those of DG. We have previously reported on our experience on MIG for advanced GC and CDH1 mutations.^11^^,^ 12 The long-term oncologic outcomes and results from randomized trials are awaited for LGT.^13^^–^ 17

Traditionally, a Roux-en-Y esophagojejunostomy is performed to reconstruct the alimentary tract after TLG. Complaints of reflux, weight loss, and dumping syndrome are reported frequently after this major surgery.^18^ There are many techniques to create a gastric reservoir,^19^ the jejunal pouch being the most common.^19^ A jejunal pouch reduces some postoperative symptoms, has less postoperative weight-loss, and although data is limited, it also improves the quality of life on a the long-term basis.^19^^–^ 21 However, only 17 % of surgeons perform a jejunal pouch reconstruction during total gastrectomy.^22^

The description of a procedure in a step-wise approach has been used by many groups as a way of standardizing the technique and facilitate learning by other surgeons, fellows, or residents.^23^^–^ 26 To further increase the uptake of TLG with jejunal pouch reconstruction, our objective was to describe a clear and thorough stepwise approach to TLG with jejunal pouch reconstruction and report our experience.

## Materials and Methods

A consensus meeting was held for technique standardization of the LTG with pouch reconstruction in a stepwise fashion. A total of 10 steps were identified. A clear description of the steps was made and approved by all the authors. Based on these steps, a didactic video was created (supplement).

From May 2007 to August 2015 all patients in the University Medical Center Utrecht who underwent LTG with jejunal pouch reconstruction for GC with curative intent were included. All patients were discussed in a multidisciplinary team meeting, prior to treatment, that included surgical oncologist, medical oncologists, radiation oncologists, gastroenterologist, radiologists, geneticists, and nutritionists. Patients’ baseline characteristics, intraoperative and postoperative data were collected in a prospectively maintained database. Complications were graded according to the Clavien-Dindo classification.^27^ Institutional Review Board approval was obtained and informed consent requirement was waived.

All statistical analyses were performed with SPSS 21.0 software (SPSS, Chicago, IL, USA). In the learning curve groups, categorical variables were compared with the Fisher’s exact test and continuous variables were analyzed with the Mann-Whitney *U* test.

## Procedure

### Step 1: Port Placement and General Inspection

**Fig. 1:**
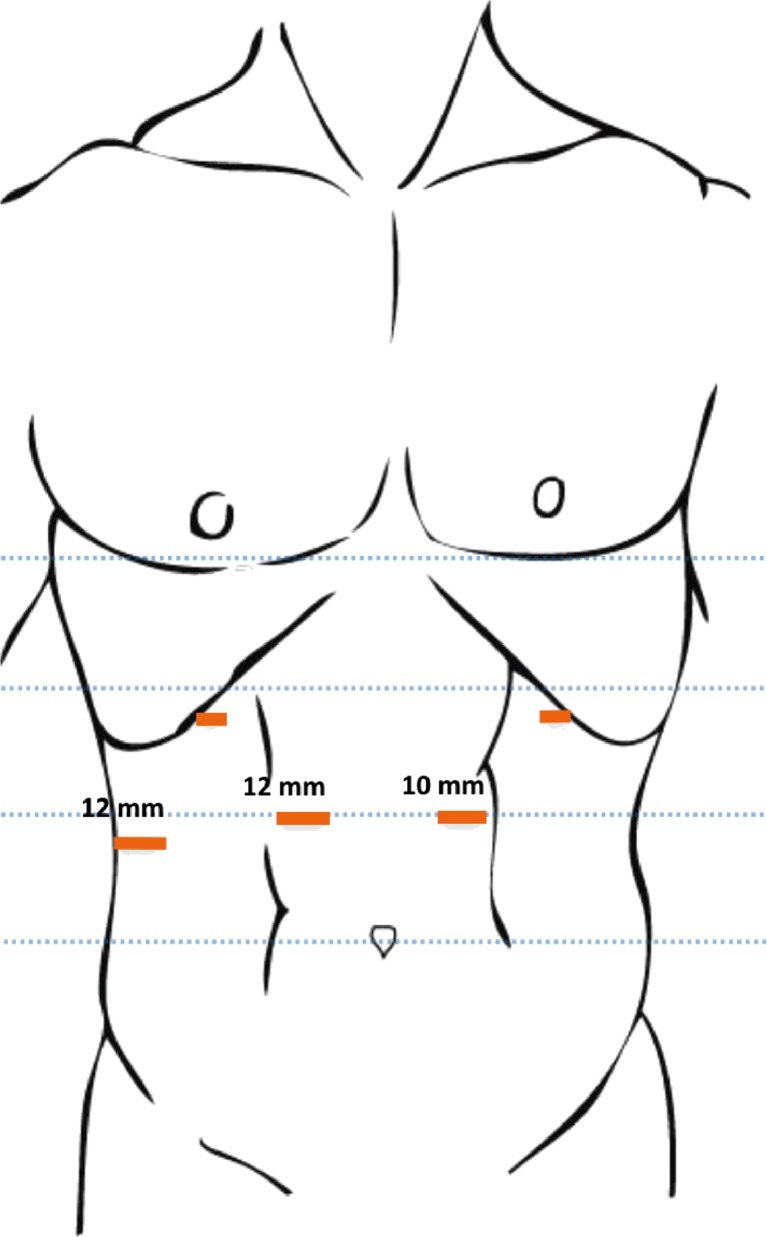
Port Placements

The patient is placed in supine position, with both arms straight against the torso. For port placement, a line is drawn from the umbilicus to the xiphoid, which is divided into three equal parts. The 10-mm camera port is then put at the level of the caudal third part, 2–4 cm from the midline, and the open Hasson technique is performed to establish pneumoperitoneum (Fig. 1).

On the contralateral side, at the same level, 3–4 cm from the midline a 12-mm working port is introduced under direct vision. At this time, a diagnostic laparoscopy is performed to rule out metastatic disease.

From this point on, two 5-mm working trocars are placed bilaterally at the subcostal level and the mid-clavicular lines. Finally, a 12 mm port is introduced at the right flank and used for liver retraction with the Endo Paddle Retract™ (Medtronic, Minneapolis, USA).

### Step 2: Division of Hepatogastric Ligament

The Endo Paddle Retract™ (Medtronic, Minneapolis, USA) is placed underneath the left liver lobe. We use the Endo Paddle Retract™ as it also helps us further up in the procedure, when the stomach is lifted up for retrogastric resection. Initially, the assistant retracts the stomach caudally, and the surgeon divides the hepatogastric ligament. The line of transection is below the left lobe of the liver and on top of the caudate lobe. After the hepatogastric ligament is divided, and if adequate retraction is maintained, the right crus and its white line can be visualized by the surgeon, and the dissection is continued perpendicular until the esophageal hiatus.

### Step 3: Division of Gastro-Colic Omentum and Short Gastric Vessels

The patient is positioned in a slight reverse Trendelenburg, which allows the transverse colon to descend. An entry point to the lesser sac is identified; usually it is easier to start towards the left side of the patient. We stay on top of the transverse colon, with care not to injure the transverse mesocolon vasculature. The omentum is not divided en bloc, as we experienced that the bulk of the omentum connected to the stomach made the exposure for gastric mobilization more difficult. Thus, the omentum is resected at a later stage.

The dissection is continued towards the left upper quadrant. The surgeon’s left hand retracts the stomach towards the patients right lower quadrant, and the assistant retracts the mesocolon caudally. After dissection along the splenic flexure, a “tunnel vision” is established demonstrating the route under the short gastric vessels, with important landmarks: the posterior gastric wall at the left side, the spleen at the right side, and the retroperitoneum with the splenic artery vein and pancreas hilum at the dorsal side. The short gastric vessels are divided cautiously with the use of an energy device. The dissection is continued until the angle of His and the left crus. It is very helpful to create space between the spleen and stomach by dividing the retrogastric adhesions first. The lymph nodes along the greater curvature (stations 4sa, 4sd and 4b) are left en bloc with the specimen.

### Step 4: Division of Left Gastric

The gastrocolic omentum that is connected to the stomach after dissection of the gastrocolic ligament, is flipped anteriorly between the stomach and the liver. The Endo Paddle Retract™ (Medtronic, Minneapolis, USA) is then placed underneath the stomach to retract it upwards. At this point the pedicle of the left gastric artery and the hepatic artery node (station 8) are clearly visible. In the background, the caudate lobe and vena cava are seen. The assistant may retract the stomach and pedicle of the left gastric artery upwards through the window underneath the stomach at the level of the caudate lobe. The surgeon starts the dissection proximal to the station 8 node and continues towards the left gastric artery pedicle (station 7). Usually the left gastric vein is encountered first and divided with the coagulating device. During further dissection at this level more cranially the artery is found, which is ligated with Hem-o-lock® clips (Teleflex, Morrisville, USA).

### Step 5: Celiac Trunk and Splenic Artery Lymphadenectomy

After completing step 4, the surgeon continues further posteriorly to harvest the celiac node (station 9), and continues on the superior border of the splenic artery to obtain the splenic nodes (stations 11p and 11d). This step must be done with great care, as the splenic artery coils along its trajectory and can be easily injured. In about 62 %, a posterior gastric artery is present between the splenic artery and the posterior gastric wall, which can be divided by coagulation.^28^ Additionally, if it is an upper third tumor involving the greater curvature, the chance of lymph node involvement is around 9–20 %, and therefore lymphadenectomy of station 10 is indicated, otherwise it is not necessary to do so. There is no benefit for routine splenectomy during D2 dissections, and on the contrary there is evidence of increased morbidity.^29^^–^ 33

### Step 6: Common Hepatic Lymphadenectomy and Right Gastric Ligation

The stomach is placed again in its natural position, and retracted downwards. The Endo Paddle Retract™ (Medtronic, Minneapolis, USA), is placed under the liver again. The previously identified hepatic artery node (station 8) is found, following the superior border of the common hepatic the origin of the gastroduodenal artery and proper hepatic will be found. The dissection is continued towards the anterior aspect of the hepatoduodenal ligament to harvest station 12a nodes. Subsequently the origin of the right gastric artery is identified, and the vessel is ligated and divided.

### Step 7: Hiatal Dissection

The dissection plane along the right crus (step 2) is found again and restarted posteriorly towards the left crus until the aorta is visualized, then continued at last finalized on the anterior aspect. The pericardial lymph nodes (stations 1 and 2) are dissected en bloc with the specimen. Now the only remaining attachments of the stomach should be the esophagus and duodenum and its tributaries.

### Step 8: Duodenal Dissection and Gastric Resection

The remaining gastrocolic omentum, located towards the duodenum, is resected. During this step, the right gastroepiploic pedicle is visualized, dissected, and ligated with the use of Hem-o-locks®. The inferior and superior border of the duodenum is cleared en bloc with the inferior and superior pyloric nodes (station 5 and 6), and a retroduodenal passage is created to allow passage of the stapler. Much care should be taken not to thermally damage the thin duodenal wall at this level during station 5, 6 dissection with a coagulation device.

The pylorus is identified and the duodenum is transected 1–2 cm distal to it, we prefer to use the Endo-GIA Purple Tri-staplers™ (Medtronic, Minneapolis, USA) to create a secure sealing of the duodenal bulb. Before firing the stapler, the surgeon should always verify that the nasogastric or nasojejunal (feeding) tube has been removed.

The stomach is then retracted caudally, and just above the site of the future proximal transection, two stay sutures are placed, one on each side of the esophagus. These are placed to avoid retraction of the esophageal stump into the thorax and control the stump during the anastomosis. With suturing, the camera is placed in the opposite 12-mm port at the right side of the patient switching with the needle driver to allow for sufficient space and angulation during suturing. Again an Endo-GIA Purple Tri-stapler ™ (Medtronic, Minneapolis, USA) is used to divide the esophagus or the proximal stomach.

### Step 9: Frozen section and Greater Omentectomy

A muscle sparing transverse incision of 3–5 cm is made at the level of the 10 mm camera port and an Endopath Dextrus™ (Ethicon, Cincinnati, USA) is inserted in order to extract the stomach through it. This Dextrus allows temporarily closure of the wound with a seal to continue the laparoscopic procedure. The specimen is sent for a frozen section of both margins, as recommended by many authors and guidelines.^34^^–^ 36

In the meantime, we proceed by completing the greater omentectomy. The omentum is retracted cephallad, and resected initially from the right side of the patient from the transverse colon, and then we proceed towards the patient’s left.

Following the current American guidelines on oncologic gastric resection, an omentectomy is performed in all cases.^37^ Although some papers and guidelines argue that omentectomy can be omitted in early gastric cancer,^34^^,^ 38 we have found it can harbor lymph nodes or milky spots in up to 18 % of the patients, and if microscopic tumor deposits in the omentum are seen in the pathology report, these patients will develop overt peritoneal carcinomatosis.^39^

### Step 10: Reconstruction

The camera port is now inserted in the Dextrus port. Once tumor involvement of the proximal transection line has been ruled out, a 25-mm OrVil™ anvil connected to a gastric tube (Medtronic, Minneapolis, USA) is passed trans-orally. The tube is pushed into the distal esophageal stump. Once it has been visualized, the esophagus is incised with the cautery and the OrVil™ (Medtronic, Minneapolis, USA) tube is extracted through the right 12 mm port until the Anvil itself is seen. The suture is cut to un-tilt the Anvil, and the tube is disconnected from it. A purse-string suture (Mersilene 3-0) is placed around the anvil to secure it, and to prevent the retraction of the esophageal mucosa, increasing the possibility of obtaining complete donuts during the anastomosis. We use suture material with a different color, so the purse-string suture is not confused with the stay sutures.

Then the ligament of Treitz is identified and 20–30 cm from it a loop of jejunal bowel is selected that is freely mobile and able to reach the site of the future Esophago-Jejunal Anastomosis (EJA). This bowel loop is extracted through the Endopath Dextrus™ (Ethicon, Cincinnati, USA), and the bowel is divided with the GIA Stapler™ (Medtronic, Minneapolis, USA). We favor the use of the 100-mm stapler size, as it is the same one we use for the creation of the jejunal pouch and its use reduces costs.

**Fig. 2:**
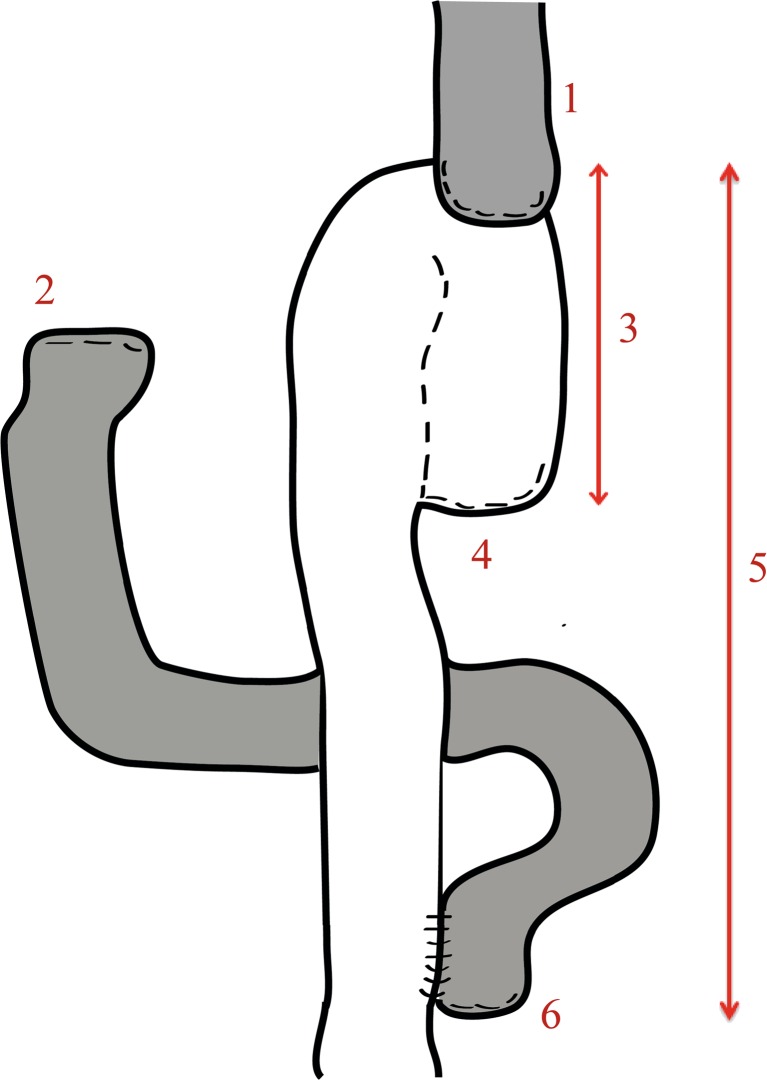
Jejunal pouch; esophagojejunostomy (*1*) dudodenal stump (*2*) jejunal J-pouch of 10 cm (*3*), blind limb (*4*), Roux-limb of 50 cm (*5*), and jejunojejunostomy (*6*)

Once the jejunum is divided, we proceed with the extracorporeal creation of the jejunal pouch (Fig. 2), which is made at the site of the already divided distal jejunum. The length of the pouch is approximately 10 cm, for which we use the 100 mm GIA Stapler™ (Medtronic, Minneapolis, USA). The bowel is folded upon itself, an enterostomy is performed on both sides of the future pouch, and then the stapler is fired to create the pouch. The enterostomy is closed with 3-0 PDS (Ethicon, Cincinnati, USA).

Next, the Roux-Y jejuno-jejunostomy is created with a side-to-side hand-sewn anastomosis of a 2 cm diameter with a continuous 3-0 PDS (Ethicon, Cincinnati, USA), approximately 40–50 cm from the site of the future EJA.

We place two stay sutures on the blind limb of the jejunal pouch, and then proceed to remove the staple line of this blind limb, the circular Orvil™ stapler will be introduced through it. These stay sutures enabled us to give counter traction on the jejunal pouch in order to facilitate correct placement of the stapler.

We then put all bowel back inside the patient’s abdomen, close the Endopath Dextrus™ (Ethicon, Cincinnati, USA) around the circular staple, and proceed to create an ante-colic EJA laparoscopically. At this point, it is important to make sure that the orange-colored base of the pin at the stapler is completely exposed and stabilize the base of the anvil with a hooked laparoscopic clamp during the connection of the anvil to the circular stapler.

After firing the circular stapler, the donuts are checked for integrity, and we proceed with closure of the abdominal incisions with PDS 1 (Ethicon, Cincinnati, USA).

## Results

### Patient Characteristics

We identified a total of 60 patients who underwent TLG; however, in 13 patients we omitted a jejunal pouch as the location of the anastomosis would be intrathoracic. A total of 47 patients had a TLG with a jejunal pouch. The majority of our patients were male (53 %) and had an American Society of Anesthesiologists (ASA) score of 2 (62 %). Median age was 66 (28–85) years. Most patients were diagnosed with advanced gastric cancer (79 %, tumor stage II-III) and underwent neo-adjuvant chemotherapy with the MAGIC protocol (3) (72 %) (Table 1).

**Table 1:** Baseline characteristics of 47 patients with jejunal pouch reconstruction after total gastrectomy

	*n*/median	(%/range)
Gender		
Male	25	(53)
Female	22	(47)
Age (years)	66	(28–85)
BMI (kg/m^2^)	23.1	(18.7–32.4)
ASA score		
1	13	(28)
2	29	(62)
3	5	(11)
Neoadjuvant therapy	34	(74)
pT-stage^a^		
T0	4	(9)
T1	2	(4)
T2	7	(15)
T3	25	(53)
T4	9	(19)
pN-stage^a^		
N0	19	(40)
N1	14	(26)
N2	4	(9)
N3	10	(21)
Tumor stage^a^		
No residual tumor	4	(9)
I	6	(13)
II	20	(43)
III	17	(36)
Harvested lymph nodes	19	(2–62)
Radicality rate (R0)	43	(91 %)

^a^Tumors were classified according to the American Joint Committee on Cancer (AJCC/)/TNM system

### Perioperative Course

Median operation time was 301 (148–454) minutes and median blood loss was 300 (30–900) milliliters (Table 2). In five (11 %) procedures, conversion to an open procedure occurred: one due to intra-operative bleeding and four due to advanced tumors with invasion of surrounding structures. EJA leakage occurred in 6/47 (12.8 %) patients, and 1/47 (2.12 %) had a leak from the pouch staple line (Table 3). Median length of stay was 11 (6–70) days, and two (4 %) patients were readmitted. With our technique, we achieved a radical resection (R0) rate of 91 % and harvested a median of 19 (range 2–62) lymph nodes.

**Table 2:** Perioperative results

Variable	*n*/median	(%/range)
Operation time (min)	301	(148–454)
Blood loss (ml)	300	(30–900)
Conversions	5	(11)
Postoperative complications	24	(51)
Anastomotic leakage	7	(15)
Pneumonia	7	(15)
Wound infection	2	(4)
Clavien-Dindo		
Grade I	1	(2)
Grade II	12	(26)
Grade III	5	(11)
Grade IV	3	(7)
In-hospital mortality	3	(6)
Hospital stay (days)	11	(6–70)
Readmissions <30 days	2	(4)

**Table 3:** Cases of anastomotic leakage

No.	Surgeon/rank^^^	Year of Surgery	Tumor stage	Age	ASA	Location of the leak	Donut	Management	Clavien-Dindo
1	1/4	2010	IIA	47	2	EJ^#^	Incomplete	Surgical	IV
2	2/1	2011	IIA	73	2	EJ^#^	Complete	Radiological	III
3	1/17	2012	IIA	66	3	EJ^#^	Complete	Surgical	V
4	2/8	2013	IIIB	81	2	EJ ^#^	Complete	Surgical	V
5	2/10	2013	CR	68	1	Pouch	Complete	Surgical	IV
6	2/12	2013	IIB	80	2	EJ^#^	Complete	Surgical	V
7	2/16	2014	IIIC	47	3	EJ^#^	Incomplete	Surgical	III

^^^Procedure rank per surgeon, ^#^esophagojejunostomy

*CR* complete response

### Long-Term Outcomes

The median follow-up of all patients was 16 (2–75) months. In the first year following surgery, the body weight of the patients decreased significantly to 87 % of the original weight (*p* < 0.001). EJA strictures occurred in 11/44 (25 %) patients, undergoing a median of three (1–5) dilatations.

### Technical Modifications

Throughout the years the following technical modifications were made:

- Omitting the jejunal pouch in case of a high esophagojejunostomy to prevent tension on the anastomosis which results in a higher risk for anastomotic leakage.
- Placement of two stay sutures on the lateral sides of the esophagus before transection in order to (a) prevent the esophageal stump to retract in the thorax, (b) control the placement of the anvil, and (c) guide the esophagus when performing the anastomosis.
- Placement of a purse-string suture around the anvil to prevent the esophageal mucosa from slipping away. With the purse-string suture, a high percentage of complete tissue donuts was achieved.
- To prevent confusion of the purse string and stay sutures, different material was used after the initial cases.
- Placement of two stay sutures on the blind limb of the jejunal pouch through which the circular stapler is introduced. These stay sutures enabled us to give counter traction on the jejunal pouch in order to facilitate correct placement of the stapler.
- Taking extra caution after unscrewing the distal button of the circular stapler, since it has the tendency to turn backwards.
- Making the jejuno-jejunostomy before the esophago-jejunostomy. In the initial procedures, the jejuno-jejunostomy was made last. It was then difficult to identify the duodenal end.

## Discussion

A step-wise approach has been described previously for many procedures, ranging from such as laparoscopic cholecystectomies,^25^^,^ 26 laparoscopic liver resections,^23^ pancreaticoduodenectomies,^24^ and laparoscopic distal gastrectomy.^40^ It is a valuable educational tool that can be used to standardize techniques, and also as a platform for teaching. TLG has been recognized as a technically demanding procedure, with a learning curve of approximately 45 cases,^41^ and to our knowledge, this is the first attempt to create a standardized step by step approach for a D2 TLG with a jejunal pouch reconstruction.

In the Netherlands, there has been an increased uptake of MIG in recent years for GC from 7.1 % of all GC surgeries in 2011 to 41 % in 2014.^42^ This can be partially explained by the centralization of GC since 2012 and a Nationwide MIG course that has been organized at our institution since 2012. This course has been recently adopted as an official European Society of Surgical Oncology Course (ESSO).^43^ The course program includes lectures that review the anatomy, operative technique and perioperative management for MIG, as well as a hands-on practice. After the course, we offer the possibility of expert proctoring in some of the first cases, this has been highly valued by former course attendees, and is one of many proven strategies to increase the uptake of minimally invasive surgery.^44^^–^ 46 We believe that a strategy including a standardized step-wise approach, a well-designed practical course and expert proctoring have all been vital to increase the uptake of MIG in the Netherlands, and can be used as an example for other countries.

The stepwise approach may help guide the surgeon more easily through the procedure, evaluate of other surgeon’s performance, or be used as a self-evaluation tool. Using these steps as a framework, a critical intraoperative time analysis may be performed, pinpointing which steps are more difficult and time-consuming. We plan on evaluating ourselves, and other surgeons time to complete the steps, as a way to look for specific strategies to improve the efficiency of the procedure, surgeon’s technique, calculate appropriate operating room times and the procedure costs.^25^^,^ 26

The anastomotic leak rate in our series is similar to the one found in the literature of 2–11 %;^20^^,^ 42 however, as we have gained more experience, our leak rate has decreased, having only 1/15 (7 %) anastomotic leakage in the last 2 years (2014/2015). We modified our technique after we had two (4 %) patients with an incomplete donut after formation of the esophagojejunostomy, both had the anastomosis reinforced with additional sutures, nevertheless, they developed a leak. This prompted us to perform two changes to our technique; the purse-string suture around the anvil and that in case of an incomplete donut we now disconnect and re-do the anastomosis all over again. We considered using a linear stapler for the anastomosis and believe it to be a fine technique as well, but we prefer the circular stapler technique because it is more suitable in case of a high anastomosis (intrathoracic) requiring more jejunal length. An additional improvement to our technique could be the development of a larger (28 mm) stapler, to reduce the anastomotic stricture rate. Until now, the 25-mm stapler is the largest available stapler. Our median hospital stay was 11, which similar to that found in other studies from western literature that focused only on total gastrectomies;^47^^,^ 48 however, we believe the recent adaptation of an ERAS protocol this median stay can be improved significantly.

We believe in the benefit of performing a jejunal pouch reconstruction, as there is evidence to suggest that it diminishes postoperative dumping and increases the patient’s quality of life in the long term.^20^^,^ 21 To our knowledge, the only MIG trial that is including some patients with pouch reconstruction is the LOGICA trial,^13^ most of them being patients from our center. We are in the process of gathering long-term quality of life date on these patients. However, we highlight the importance of this technique allows the benefits of the MIG approach with the jejunal pouch reconstruction.

## Conclusion

Laparoscopic total gastrectomy with jejunal pouch reconstruction demonstrated to be a feasible procedure in a selected group of patients with good perioperative outcomes for western patients. Our step-wise approach might help surgeons to introduce laparoscopic total gastrectomy with jejunal pouch reconstruction in their center. Additionally this is will create a framework for us to continue teaching the procedure, and also evaluate future surgeons learning the procedure.

## Electronic supplementary material

Below is the link to the electronic supplementary material.

ESM 1 (MP4 316757 kb)

## References

[1] Ferro A, Peleteiro B, Malvezzi M, Bosetti C, Bertuccio P, Levi F (2014). Worldwide trends in gastric cancer mortality (1980-2011), with predictions to 2015, and incidence by subtype. Eur J Cancer.

[2] Ferlay J, Soerjomataram I, Dikshit R, Eser S, Mathers C, Rebelo M (2015). Cancer incidence and mortality worldwide: sources, methods and major patterns in GLOBOCAN 2012. Int J Cancer.

[3] Cunningham D, Allum WH, Stenning SP, Thompson JN, Van de Velde CJ, Nicolson M (2006). Perioperative chemotherapy versus surgery alone for resectable gastroesophageal cancer. N Engl J Med.

[4] Kim W, Kim HH, Han SU, Kim MC, Hyung WJ, Ryu SW (2016). Decreased morbidity of laparoscopic distal gastrectomy compared with open distal gastrectomy for stage I gastric cancer: short-term outcomes from a multicenter randomized controlled trial (KLASS-01). Ann Surg.

[5] Jiang L, Yang KH, Guan QL, Cao N, Chen Y, Zhao P (2013). Laparoscopy-assisted gastrectomy versus open gastrectomy for resectable gastric cancer: an update meta-analysis based on randomized controlled trials. Surg Endosc.

[6] Vinuela EF, Gonen M, Brennan MF, Coit DG, Strong VE (2012). Laparoscopic versus open distal gastrectomy for gastric cancer: a meta-analysis of randomized controlled trials and high-quality nonrandomized studies. Ann Surg.

[7] Best LM, Mughal M, Gurusamy KS (2016). Laparoscopic versus open gastrectomy for gastric cancer. Cochrane Database Syst Rev..

[8] Hu Y, Huang C, Sun Y, Su X, Cao H, Hu J (2016). Morbidity and mortality of laparoscopic versus open D2 distal gastrectomy for advanced gastric cancer: a randomized controlled trial. J Clin Oncol.

[9] Haverkamp L, Weijs TJ, van der Sluis PC, van der Tweel I, Ruurda JP, van Hillegersberg R (2013). Laparoscopic total gastrectomy versus open total gastrectomy for cancer: a systematic review and meta-analysis. Surg Endosc.

[10] Chen K, Pan Y, Cai JQ, Xu XW, Wu D, Mou YP (2014). Totally laparoscopic gastrectomy for gastric cancer: a systematic review and meta-analysis of outcomes compared with open surgery. World J Gastroenterol.

[11] Haverkamp L, Ruurda JP, Offerhaus GJ, Weijs TJ, van der Sluis PC, van Hillegersberg R (2016). Laparoscopic gastrectomy in Western European patients with advanced gastric cancer. Eur J Surg Oncol.

[12] Haverkamp L, van der Sluis PC, Ausems MG, van der Horst S, Siersema PD, Ruurda JP (2015). Prophylactic laparoscopic total gastrectomy with jejunal pouch reconstruction in patients carrying a CDH1 germline mutation. J Gastrointest Surg.

[13] Haverkamp L, Brenkman HJ, Seesing MF, Gisbertz SS, van Berge Henegouwen MI, Luyer MD (2015). Laparoscopic versus open gastrectomy for gastric cancer, a multicenter prospectively randomized controlled trial (LOGICA-trial). BMC Cancer..

[14] Straatman J, van der Wielen N, Cuesta MA, Gisbertz SS, Hartemink KJ, Alonso Poza A (2015). Surgical techniques, open versus minimally invasive gastrectomy after chemotherapy (STOMACH trial): study protocol for a randomized controlled trial. Trials..

[15] Hur H, Lee HY, Lee HJ, Kim MC, Hyung WJ, Park YK (2015). Efficacy of laparoscopic subtotal gastrectomy with D2 lymphadenectomy for locally advanced gastric cancer: the protocol of the KLASS-02 multicenter randomized controlled clinical trial. BMC Cancer..

[16] Kim HH, Han SU, Kim MC, Hyung WJ, Kim W, Lee HJ (2013). Prospective randomized controlled trial (phase III) to comparing laparoscopic distal gastrectomy with open distal gastrectomy for gastric adenocarcinoma (KLASS 01). J Korean Surg Soc.

[17] Laparoscopy-assisted Total Gastrectomy for Clinical Stage I Gastric Cancer (KLASS-03) [Internet]. Available from: https://clinicaltrials.gov/ct2/show/NCT01584336.

[18] Liedman B (1999). Symptoms after total gastrectomy on food intake, body composition, bone metabolism, and quality of life in gastric cancer patients--is reconstruction with a reservoir worthwhile?. Nutrition.

[19] Lehnert T, Buhl K (2004). Techniques of reconstruction after total gastrectomy for cancer. Br J Surg.

[20] Fein M, Fuchs KH, Thalheimer A, Freys SM, Heimbucher J, Thiede A (2008). Long-term benefits of Roux-en-Y pouch reconstruction after total gastrectomy: a randomized trial. Ann Surg.

[21] Gertler R, Rosenberg R, Feith M, Schuster T, Friess H (2009). Pouch vs. no pouch following total gastrectomy: meta-analysis and systematic review. Am J Gastroenterol.

[22] Brenkman HJF HL, Ruurda JP, van Hillegersberg R. Worldwide practice in gastric cancer surgery. World J Gastroenterol. 2016; 22(15):4041-8. 10.3748/wjg.v22.i15.4041 PMC4823255 27099448

[23] Toro JP, Patel AD, Lytle NW, Sweeney JF, Medbery RL, Scott Davis S (2015). Detecting performance variance in complex surgical procedures: analysis of a step-wise technique for laparoscopic right hepatectomy. Am J Surg.

[24] Marangoni G, Morris-Stiff G, Deshmukh S, Hakeem A, Smith AM (2012). A modern approach to teaching pancreatic surgery: stepwise pancreatoduodenectomy for trainees. J Gastrointest Surg.

[25] Traverso LW, Koo KP, Hargrave K, Unger SW, Roush TS, Swanstrom LL (1997). Standardizing laparoscopic procedure time and determining the effect of patient age/gender and presence or absence of surgical residents during operation. A prospective multicenter trial. Surg Endosc.

[26] Berber E, Engle KL, Garland A, String A, Foroutani A, Pearl JM (2001). A critical analysis of intraoperative time utilization in laparoscopic cholecystectomy. Surg Endosc.

[27] Dindo D, Demartines N, Clavien PA (2004). Classification of surgical complications: a new proposal with evaluation in a cohort of 6336 patients and results of a survey. Ann Surg.

[28] Suzuki K, Prates JC, DiDio LJ (1978). Incidence and surgical importance of the posterior gastric artery. Ann Surg.

[29] Degiuli M, De Manzoni G, Di Leo A, D’Ugo D, Galasso E, Marrelli D (2016). Gastric cancer: current status of lymph node dissection. World J Gastroenterol.

[30] Brar SS, Seevaratnam R, Cardoso R, Law C, Helyer L, Coburn N (2012). A systematic review of spleen and pancreas preservation in extended lymphadenectomy for gastric cancer. Gastric Cancer..

[31] Sano T. Randomized controlled trial to evaluate splenectomy in total gastrectomy for proximal gastric carcinoma (JCOG0110): final survival analysis. 2015 Gastrointestinal Cancers Symposium; Chicago, IL, USA2015.

[32] Csendes A, Burdiles P, Rojas J, Braghetto I, Diaz JC, Maluenda F (2002). A prospective randomized study comparing D2 total gastrectomy versus D2 total gastrectomy plus splenectomy in 187 patients with gastric carcinoma. Surgery.

[33] Yu W, Choi GS, Chung HY (2006). Randomized clinical trial of splenectomy versus splenic preservation in patients with proximal gastric cancer. Br J Surg.

[34] Japanese Gastric Cancer A (2011). Japanese gastric cancer treatment guidelines 2010 (ver. 3). Gastric Cancer..

[35] Squires MH, Kooby DA, Pawlik TM, Weber SM, Poultsides G, Schmidt C (2014). Utility of the proximal margin frozen section for resection of gastric adenocarcinoma: a 7-Institution Study of the US Gastric Cancer Collaborative. Ann Surg Oncol.

[36] Chen JD, Yang XP, Shen JG, Hu WX, Yuan XM, Wang LB (2013). Prognostic improvement of reexcision for positive resection margins in patients with advanced gastric cancer. Eur J Surg Oncol.

[37] Ajani JA, Bentrem DJ, Besh S, D’Amico TA, Das P, Denlinger C (2013). Gastric cancer, version 2.2013: featured updates to the NCCN Guidelines. J Natl Compr Canc Netw.

[38] Ha TK, An JY, Youn HG, Noh JH, Sohn TS, Kim S (2008). Omentum-preserving gastrectomy for early gastric cancer. World J Surg.

[39] Haverkamp L, Brenkman HJ, Ruurda JP, Ten Kate FJ, van Hillegersberg R. The oncological value of omentectomy in gastrectomy for cancer. J Gastrointest Surg. 2016. 10.1007/s11605-016-3092-4 PMC4850186 26895951

[40] Ben-David K, Tuttle R, Kukar M, Oxenberg J, Hochwald SN (2015). Laparoscopic distal, subtotal gastrectomy for advanced gastric cancer. J Gastrointest Surg.

[41] Jeong O, Ryu SY, Choi WY, Piao Z, Park YK (2014). Risk factors and learning curve associated with postoperative morbidity of laparoscopic total gastrectomy for gastric carcinoma. Ann Surg Oncol.

[42] (DICA) DIfCA. Gastric and Esophageal Cancer Yearly Report 2014 [Available from: http://www.clinicalaudit.nl/jaarrapportage/2014/duca.html.

[43] Oncology ESS. ESSO Courses and Masterclasses [Available from: http://www.essoweb.org/eursso/education/courses-a-masterclasses-more-info-here.html.

[44] Moloo H, Haggar F, Martel G, Grimshaw J, Coyle D, Graham ID (2009). The adoption of laparoscopic colorectal surgery: a national survey of general surgeons. Can J Surg.

[45] Dominguez EP, Barrat C, Shaffer L, Gruner R, Whisler D, Taylor P (2013). Minimally invasive surgery adoption into an established surgical practice: impact of a fellowship-trained colleague. Surg Endosc.

[46] Birch DW, Asiri AH, de Gara CJ (2007). The impact of a formal mentoring program for minimally invasive surgery on surgeon practice and patient outcomes. Am J Surg.

[47] Papenfuss WA, Kukar M, Oxenberg J, Attwood K, Nurkin S, Malhotra U (2014). Morbidity and mortality associated with gastrectomy for gastric cancer. Ann Surg Oncol.

[48] Topal B, Leys E, Ectors N, Aerts R, Penninckx F (2008). Determinants of complications and adequacy of surgical resection in laparoscopic versus open total gastrectomy for adenocarcinoma. Surg Endosc.

